# The Impact of Trimetazidine on Cardiac Fibrosis, Inflammation, and Function in Ischemic Cardiomyopathy Patients

**DOI:** 10.1007/s10557-022-07340-0

**Published:** 2022-05-10

**Authors:** Noha M. El-khodary, Asser I. Ghoneim, Ahmed A. El-tayaar, Eman M. El-touny

**Affiliations:** 1 Department of Clinical Pharmacy, Faculty of Pharmacy, Kafrelsheikh University, Kafrelsheikh City, Egypt; 2 Department of Pharmacology & Toxicology, Faculty of Pharmacy, Damanhour University, Damanhour City, Egypt; 3 Department of Internal Medicine & Cardiology, Damanhour National Medical Instititue, Damanhour City, Egypt; 4 Department of Clinical Pharmacy & Pharmacy Practice, Faculty of Pharmacy, Damanhour University, Damanhour City, Egypt

**Keywords:** Trimetazidine, ischemic cardiomyopathy, vascular endothelial function, inflammatory factors, cardiac fibrosis

## Abstract

**Background:**

Previous studies have shown that Trimetazidine (TMZ) improves vascular endothelial function and reduces the inflammatory process progression. However, limited data have been available regarding its effects on myocardial fibrosis following ischemia and causing left ventricular dysfunction.

**Purpose:**

To investigate the impact of TMZ adjuvant therapy for ischemic cardiomyopathy (ICM) on cardiac fibrosis, vascular endothelial function, inflammation, and myocardial functions.

**Methods:**

This randomized, double-blind controlled clinical trial included 48 patients (aged 59.4 ± 9 years) with ICM who were randomly assigned to two groups: TMZ 35 mg twice daily and placebo in addition to conventional ICM medications. All patients received the tablets for 3 months. Both groups were then compared in terms of connective tissue growth factor (CTGF), endothelin-1 (ET-1), tumor necrosis factor-alpha (TNF-α), and some echocardiographic indices, weekly angina attacks and nitrate consumption before and after treatment.

**Results:**

No significant differences between CTGF, ET-1, and TNF-α levels, in addition to some echocardiographic indices, were observed between both groups before treatment. After treatment, the TMZ group had significantly lower ET-1 than the placebo group, with both groups exhibiting a substantial decrease in TNF-α and CTGF. The TMZ group had lower mean ± SD levels for TNF-α and CTGF and showed significant improvement in echocardiographic indices and weekly angina attacks after treatment.

**Conclusion:**

Adjunctive TMZ therapy for ICM effectively improved vascular endothelial function and reduced inflammation. Furthermore, our exploratory findings may be used to provide new information on the potential effects of TMZ on myocardial fibrosis by downregulating CTGF.

## Introduction

Coronary artery disease (CAD) is one of the most common cardiovascular disorders affecting people worldwide. In both developed and developing countries, this disease has been proven to be the leading cause of death [1]. Ischemic cardiomyopathy (ICM), a term that refers to heart muscle weakness resulting from ischemia or heart attack, which leads to decreased ability of the heart to pump blood [2], has been considered the most severe complication of coronary heart disease [3]. ICM can be characterized as a left ventricular (LV) function deficiency caused by CAD, which results in increased mortality rates and poor quality of life. LV dysfunction in ICM patients may be caused by necrosis or myocardial fibrosis [4]. The most common non-modifiable risk factors for ICM include old age, male sex, and family history. On the other hand, hypertension, diabetes, hyperlipidemia, obesity, smoking, and sedentary lifestyle are the most important modifiable risk factors for ICM [2]. While receiving conventional medication, a large number of ICM patients tend to experience symptoms. Therefore, there is a need to include other ICM drugs with different mechanisms of action [4].

Reports have shown that Trimetazidine (TMZ), a dihydrochloride of 2,3,4-trimethoxybenzyl-piperazine, exerts anti-ischemic properties without any effect on myocardial oxygen intake or blood supply [5]. Some trials have demonstrated that TMZ is beneficial for patients who suffer from heart failure [5]. TMZ is a cardioprotective agent given that it alters energy substrate metabolism. By inhibiting the 3-ketoacyl-coenzyme A thiolase enzyme, TMZ reduces long-chain fatty acid oxidation. This reduction in fatty acid oxidation contributes to a decrease in oxygen intake, which is very beneficial in ischemic conditions, and increases the activity of the pyruvate dehydrogenase enzyme, which contributes to increased glucose oxidation. The homeostasis between glycolysis and glucose oxidation improved following changes in the energy substrate, leading to a reduction in proton and Na+ accumulation. The net result is the more efficient generation of adenosine triphosphate (ATP) in a low-oxygen environment [6, 7]. TMZ is believed to directly suppress cardiac fibrosis. In fact, evidence has shown that TMZ decreases collagen aggregation, connective tissue growth factor (CTGF) expression in cardiac fibroblasts, nicotinamide adenine dinucleotide phosphate-oxidase levels, and production of reactive oxygen species, with the positive impact of this drug on the treatment of congestive heart failure having been attributed to the mentioned mechanisms [6, 8]. Owing to decreased serum endothelin-1 (ET-1) levels, TMZ enhances myocardial endothelial function, thereby alleviating myocardial damage and chronic myocardial ischemia [9]. TMZ reduces the level of oxidative stress markers, including malondialdehyde (MDA), the end product of lipid peroxidation, by reducing membrane injury triggered by reactive oxygen species [10]. TMZ reduces the release of proinflammatory mediators from macrophages induced by reactive oxygen species, including C-reactive protein (CRP), tumor necrosis factor-alpha (TNF-α), interleukin 1 (IL-1), and interleukin 8 (IL-8) during both inflammation and ischemia [10].

CTGF is a member of the CCN family of multifunctional matricellular proteins that contribute to the initiation of fibrosis in a variety of organs and tissues, including the heart. CTGF is also a key mediator of the profibrotic cytokine transforming growth factor- (TGF-) signaling pathway. Several molecules, such as TGF-, Smads, CTGF, corin, mesenchymal cell products, and inflammatory agents, influence the fibrosis process and collagen metabolism. CTGF stimulates the proliferation of fibroblast and increases the content of the extracellular matrix. CTGF is a “bystander” marker that has the potential to indirectly influence the fibrosis process [11]. However, only a few studies have assessed CTGF, all of which have been in animals [12, 13]. Hence, no clinical trial has yet investigated CTGF inhibition for the treatment of heart diseases [14].

Previous studies have documented the benefits of TMZ in ICM in terms of vascular endothelial function, inflammatory process, and myocardial functions. Thus, the current research aimed to investigate whether TMZ, as an adjuvant drug to conventional ICM therapies, affects cardiac fibrosis assessed using CTGF expression and determine the clinical benefits of adding TMZ on vascular endothelial function, inflammatory process, and myocardial function. The current study has been the first to evaluate CTGF in clinical settings. The assessment of CTGF as an initiator biomarker of cardiac fibrosis [11] will be beneficial for NYHA II patients who are at the early stage of disease progression.

## Methods

### Patients

This randomized, double-blind, controlled clinical trial was conducted on 48 patients with ICM aged between 34 and 80 years recruited from the Cardiology Clinic at Damanhour National Medical Institute from March 2019 to February 2020. All patients agreed to participate in this clinical study and provided informed consent. The study was approved by the Research Ethics Committee of the Faculty of Pharmacy, Damanhour University (No: 1218PP9), and the Ethics Committee of Damanhour National Medical Institute in accordance with the Declaration of Helsinki and its amendments.

### Inclusion Criteria

Patients were included when they satisfied the following inclusion criteria: (1) should have adequate hematological functions and normal complete blood counts and coagulation profile (PT, activity, aPTT, and INR); (2) should have normal liver, kidney, and thyroid functions; (3) should have a history of CAD; (4) LV ejection fraction (LVEF) ≤40%; (5) cardiac function characterized as NYHA grade II; and (6) age between 34 and 80 years.

### Exclusion Criteria

Patients who satisfied the following inclusion criteria were excluded: (1) serious disorders of the liver, such as hepatitis, hemochromatosis, and Wilson disease; (2) chronic kidney disease or acute kidney injury (AKI); (3) serious brain disorders, such as previous stroke, epilepsy, dementia, and Alzheimer’s disease; (4) malignant tumors and autoimmune diseases; (5) cardiogenic shock in patients with decompensated heart failure; (6) systolic blood pressure <100 mmHg (baseline blood pressure was measured for each patient three times on three consecutive days, after which the average was obtained); (7) Parkinson’s disease or motor disorders; (8) allergies to TMZ; and (9) pregnancy and breastfeeding.

### Study Design

Patients were randomized into two groups matched for age, weight, and medication: (1) the TZM group [24 patients who received a 35-mg TMZ modified-release (MR) tablet twice daily for 3 months as defined by the European Society of Cardiology (ESC)] [15]; (2) the placebo group (24 patients who received a placebo). The placebo was similar to TMZ tablets in appearance and taste. All patients in both groups were provided conventional ICM treatments, such as antithrombotic therapy, beta-blockers, lipid-lowering therapy, angiotensin-converting enzyme inhibitors (ACEIs)/or angiotensin II receptor blockers, nitrates, and diuretics [15].

Investigators and patients were blinded to the randomization code. The participants were asked to continue their routine dietary intake, physical activity, and medication during the study period. A general information questionnaire requesting data on medication, weight, height, age, ICM duration (years), and physical activity was collected by a trained interviewer.

### Sample Collection

Before and after treatment, 3–4 mL of fasting venous blood was extracted from the patients in the morning. The samples were then left at room temperature for 10–20 min for coagulation and centrifugation at 2000–3000 revolutions per minute (r.p.m) for 20 min. Sera were collected and then placed in the refrigerator at −80 °C until analysis.

### Monitoring Indexes

The two groups were compared in terms of vascular endothelial function (assessed using ET-1), inflammatory process (assessed using TNF-α), and cardiac fibrosis (assessed using CTGF). Serum levels of ET-1, TNF-α, and CTGF were measured using enzyme-linked immunosorbent assay (ELISA) for each patient in both the study and placebo groups before and after treatment. The kit was provided by Biokit for Scientific Research Company. Echocardiographic parameters, such as LVEF, LV end-systolic diameter (LVESD), and LV end-diastolic diameter (LVEDD), were measured before and after treatment. Also, number of angina attacks and nitrate consumption per week were collected before and after treatment.

### Image Analysis

All echocardiograms were completed with color Doppler echocardiography using a PHILIPS HD 11 Echograph machine (United States). An experienced investigator analyzed the echocardiograms. Some echocardiographic indices, including LVEDD, LVESD, and LVEF were recorded and data were stored in electronic files.

### Echocardiography

LVEF was measured using the modified Simpson method (biplane method of disks). This method of LVEF measurement has been recommended by the American Society of Echocardiography. This method entails measuring LVEF at end-systole and end-diastole by tracing the endocardial border in both the apical four-chamber and two-chamber views [16].

### Statistical Analysis

The required sample size was calculated based on previously treated trial cases (E-1 was assessed) [17], after which a power analysis was conducted (G power version 3.1 statistical software, Franz Faul, Universität Kiel Germany). Differences between two independent means (two groups) were calculated to determine the required sample size given α, power, and effect size. The input parameters were an α error probability of 0.05, an effect size (f) of 0.8324939, a power of 0.80, and two groups. The findings indicated that a minimum sample size of *n* = 48 samples was required (24 samples for each group).

The IBM SPSS software package version 20.0 was used to analyze the data that were fed into the device (IBM Corporation, Armonk, NY). The Kolmogorov–Smirnov test was used to confirm whether the variables had a normal distribution. The Chi-square test (Fisher Exact correction) was used to determine differences in categorical variables between the groups; the Student *t*-test was used to compare two classes of normally distributed quantitative variables, and the paired *t*-test was used to compare two intervals of normally distributed quantitative variables. Non-normally distributed quantitative variables were compared using the Mann–Whitney test. Abnormally distributed quantitative variables were compared using the Wilcoxon signed-rank test. Correlation analysis between variables was performed using Pearson’s coefficient. The significance of the obtained results was calculated at a significance level of 5%.

## Results

**Fig. 1:**
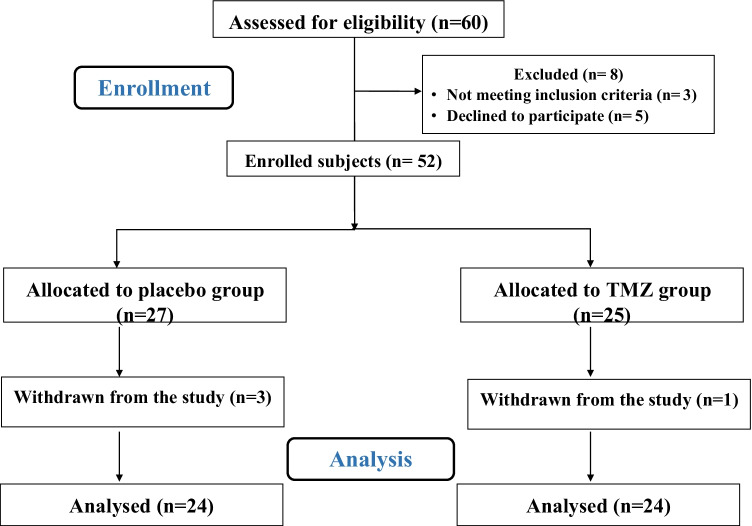
Flow diagram of the study

After screening the patients according to the inclusion and exclusion criteria, as previously described, 52 patients were initially included in this study. However, during the 3-month treatment period, four patients were excluded for the following reasons: irregular use of TMZ or for less than 3 months (one patient) and non-adherence to their medications (three patients). Finally, 48 patients completed the study as shown in Fig. 1.

The average age of the study participants was 59.4 ± 9 years, and 60.4% of the participants were males. As shown in Table 1, the two groups had almost similar baseline demographics, with no statistically significant differences. Moreover, 18.75% of the participants were smokers, 62.5% had diabetes, 47.9% had a previous history of percutaneous intervention, and 20.8% had undergone post-coronary artery bypass grafting (CABG). Baseline medications were comparable in the two groups.

**Table 1:** Baseline demographics of patients in the two groups

Parameter	TMZ group (*n* = 24)	Placebo group (*n* = 24)	**P**
Gender			
Male	15 (62.5%)	14 (58.3%)	0.768
Female	9 (37.5%)	10 (41.7%)	
Age (years)			
Min–Max	38–71	34–80	0.789
Mean ± SD	59.1 ± 9	59.8 ± 9.25	
BMI (kg/m^2^)			
Min–Max Mean ± SD	21.9–32 26.4 ± 3.1	21.8–32.8 25.6 ± 2.9	0.311
Heart rate (beats/min.)			
Min–Max	70–90	65–90	0.946
Mean ± SD	76.3 ± 4.8	76.5 ± 7.5	
Systolic blood pressure (mmHg)			
Min–Max	100–180	100–180	0.900
Mean ± SD	139.6 ± 23.2	140.4 ± 22.45	
Diastolic blood pressure (mmHg)			
Min–Max	70–110	70–110	1.000
Mean ± SD	88.3 ± 12.3	88.3 ± 12.3	
Smoking	3 (12.5%)	6 (25%)	^FE^*p*=0.461
Diabetes	14 (58.3%)	16 (66.7%)	0.551
Hyperlipidemia	11 (45.83%)	13 (54.16%)	0.563
Prior PCI			
0	13 (54.2%)	12 (50%)	0.773
1	11 (45.8%)	12 (50%)	
Prior CABG			
0	19 (79.2%)	19 (79.2%)	1.000
1	5 (20.8%)	5 (20.8%)	
Medications			
Aspirin	22 (91.7%)	24 (100%)	^FE^***P***=0.489
Clopidogrel	6 (25%)	5 (20.8%)	0.731
Statins	22 (91.7%)	23 (95.8%)	^FE^***P***=1.000
ACEIs	15 (62.5%)	14 (58.3%)	0.768
ARBs	4 (16.7%)	5 (20.8%)	^FE^***P***=1.000
Beta blockers	20 (83.3%)	21 (87.5%)	1.000
Nitrates	18 (75%)	17 (70.8%)	0.745
Diuretics	14 (58.3%)	13 (54.2%)	0.771

*FE*, Fisher exact; *t*, Student *t*-test

***P***, ***P***-value for comparing between the studied groups

### Comparison of Vascular Endothelial Function Between the Two Groups

No significant differences in ET-1 levels were observed between the TMZ and placebo groups before treatment (***P*** > 0.05). The TMZ group had a significantly lower ET-1 level after than before treatment (***P*** < 0.05). Meanwhile, the placebo group had a lower ET-1 level after than before treatment, but the difference was not statistically significant (***P*** > 0.05). The TMZ group had substantially lower ET-1 levels (39.5 ± 50.9 ng/L) than the placebo group (44.46 ± 29.14 ng/L) after treatment (***P*** < 0.05) (Table 2).

**Table 2:** Comparison of vascular endothelial function between the two groups

ET-1 (ng/L)	TMZ group (*n* = 24)	Placebo group (*n* = 24)	**P**
Baseline			
Min–Max	2.5–165.5	22.7–172.5	0.180
Mean ± SD	91.1 ± 61.5	58.8 ± 36.7	
After 3 months			
Min–Max	2.5–167.5	7.50–99.50	0.041^*^
Mean ± SD	39.5 ± 50.9	44.46 ± 29.14	
Decrease	**51.55 ± 81.9**	**14.4 ± 41.4**	0.016^*^
**P**_**1**_	0.006^*^	0.153	

**P**, ***P***-value for comparing between the two groups

***P***_1_, ***P***-value for Wilcoxon signed ranks test for comparing between before and after in each group

* Statistically significant at ***P*** ≤ 0.05

### Comparison of the Inflammatory Process Between the Two Groups

No significant difference in TNF-α levels was observed between the TMZ and placebo groups before treatment (***P*** > 0.05). The TMZ and placebo groups exhibited a significantly lower TNF-α level after than before treatment (***P*** < 0.05, ***P*** < 0.05). After treatment, the TMZ group had significantly lower TNF-α levels (82 ± 100 ng/L) than the placebo group (222 ± 186.5 ng/L) (***P*** < 0.05) (Table 3).

**Table 3:** Comparison of inflammatory process between the two groups

TNF-α (ng/L)	TMZ group (*n* = 24)	Placebo group (*n* = 24)	**P**
Baseline			
Min–Max	19–502.5	53–502.5	0.207
Mean ± SD	368.2 ± 163.5	407.7 ± 154.5	
After 3 months			
Min–Max	16.5–502.5	22.8–502.5	0.006^*^
Mean ± SD	82 ± 100	222 ± 186.5	
Decrease	**286.2 ± 192.1**	**185.7 ± 172.2**	0.044^*^
**P**_**1**_	<0.001^*^	<0.001^*^	

**P**, *P*-value for comparing between the studied groups

**P**_1_, *P*-value for Wilcoxon signed ranks test for comparing between before and after in each group

* Statistically significant at ***P*** ≤ 0.05

### Comparison of Cardiac Fibrosis Process Between the Two Groups

There were no significant differences in CTGF levels between the two groups before treatment (***P*** > 0.05), as shown in Table 4. In the TMZ group, CTGF levels were significantly lower after than before treatment (***P*** < 0.05). The placebo group had a significantly lower CTGF level after than before treatment (***P*** < 0.05). After treatment, the TMZ group had substantially lower CTGF levels (883 ± 865.8 pg/mL) compared to the placebo group (1654.6 ± 1099.1 pg/mL) (***P*** < 0.05).

**Table 4.** Comparison of cardiac fibrosis between the two groups

CTGF (pg/ml)	TMZ group (*n* = 24)	Placebo group (*n* = 24)	**P**
Baseline			
Min–Max	50–3350	177–3350	0.820
Mean ± SD.	2218.7 ± 1056.5	2330.5 ± 1020.9	
After 3 months			
Min–Max	2.6–3230	337.6–3620	0.005^*^
Mean ± SD	883 ± 865.8	1654.6 ± 1099.1	
Decrease	**1335.8 ± 1314.3**	**675.9 ± 1250.95**	0.045^*^
**P**_**1**_	<0.001^*^	0.018^*^	

**P**, ***P***-value for comparing between the studied groups

**P**_1_, ***P***-value for Wilcoxon signed ranks test for comparing between before and after in each group

* Statistically significant at **P** ≤ 0.05

### Comparison of Myocardial Function Between the Two Groups

The LVEF, LVEDD, and LVESD values showed no significant differences between the two groups before treatment (***P*** > 0.05) (Table 5). The TMZ group showed a significantly higher LVEF after than before treatment (***P*** < 0.05). After treatment, the TMZ group had significantly higher LVEF levels (38.1% ± 4.2%) than the placebo group (34.5% ± 5.6%) (***P*** < 0.05), although a significant decrease in the levels of LVEDD and LVESD was observed (***P*** < 0.05). After treatment, the TMZ group had significantly lower LVESD and LVEDD levels (44.8 ± 4.3 mm and 57.9 ± 3.4 mm, respectively) than the placebo group (48.3 ± 3.9 mm and 59.75 ± 2.7 mm, respectively) (***P*** < 0.05).

**Table 5:** Comparison of myocardial function between the two groups

		TMZ group (*n* = 24)	Placebo group (*n* = 24)	**P**
LVEF (%)	Baseline			
	Min–Max	25–40	25–40	0.196
	Mean ± SD	36.3 ± 4.4	34.5 ± 5.3	
	After 3 months			
	Min–Max	26–43	25–46	0.019^*^
	Mean ± SD	38.1 ± 4.5	34.5 ± 5.6	
	Increase	**1.8 ± 1.1**	**0 ± 2.5**	<0.001^*^
	**P**_**1**_	<0.001^*^	1.000	
LVESD (mm)	Baseline			
	Min–Max	42–55	41–55	0.562
	Mean ± SD	48.8 ± 3.3	48.2 ± 3.6	
	After 3 months			
	Min–Max	39–53	38–56	0.004^*^
	Mean ± SD	44.8 ± 4.3	48.3 ± 3.9	
	Decrease	**4 ± 2.8**	**-0.1 ± 1.1**	<0.001^*^
	**P**_**1**_	<0.001^*^	0.588	
LVEDD (mm)	Baseline			
	Min–Max	53–67	55–65	0.380
	Mean ± SD	59.2 ± 3.7	60 ± 2.7	
	After 3 months			
	Min–Max	53–66	55–66	0.043^*^
	Mean ± SD	57.9 ± 3.4	59.75 ± 2.7	
	Decrease	**1.3 ± 1.7**	**0.3 ± 2.1**	0.043^*^
	**P**_**1**_	0.002^*^	0.560	

t, Student t-test

**P**, ***P***-value for comparing between the studied groups

**P**_1_, ***P***-value for paired t-test for comparing between before and after in each group

* Statistically significant at ***P*** ≤ 0.05

Given that TMZ was well tolerated, patients did not need to stop taking it due to side effects and change the dose during the trial. No substantial change in any biochemical parameters was observed throughout the follow-up period.

### Correlation Between ET-1, CTGF, and TNF-α and Echocardiographic Indices in the TMZ Group

No significant correlation was observed between ET-1, CTGF, and TNF-α and echocardiographic indices in the TMZ group (***P*** > 0.05), as shown in Table 6.

**Table 6:** Correlation between ET-1, CTGF, TNF-α with echocardiographic indices in TMZ group (*n* = 24)

		ET-1		CTGF		TNF-α	
		Baseline	After 3 months	Baseline	After 3 months	Baseline	After 3 months
LVEF (%)	**r**	0.289	-0.146	-0.126	0.068	-0.250	0.056
	**P**	0.171	0.495	0.559	0.753	0.239	0.795
LVESD (mm)	**r**	-0.223	-0.132	0.161	0.185	0.024	0.306
	**P**	0.294	0.538	0.452	0.387	0.913	0.146
LVEDD (mm)	**r**	-0.035	-0.248	0.312	0.032	0.082	0.168
	**P**	0.869	0.242	0.137	0.881	0.703	0.434

r, Pearson coefficient

### Comparison of the Number of Angina Attacks Per Week Between the Two Groups

No significant differences in the number of angina attacks per week were observed between the two groups before treatment (***P*** > 0.05), as shown in Table 7. In the TMZ group, the number of angina attacks per week was significantly lower after than before treatment (***P*** < 0.05). The placebo group had no significant differences in the number of angina attacks per week after than before treatment (***P*** >0.05). After treatment, the TMZ group had a substantially lower number of angina attacks per week compared to the placebo group (***P*** < 0.05).

**Table 7:** Comparison of the number of angina attacks per week between the two groups

Number of angina attacks per week	TMZ group (*n* = 24)	Placebo group (*n* = 24)	**P**
Baseline			
Mean ± SD	3.1 ± 1.8	3.2 ± 1.5	0.77
After 3 months			
Mean ± SD	2.2 ± 1.3	2.9 ± 1.4	0.009^*^
**P**_**1**_	0.003^*^	0.057	

**P**, ***P***-value for comparing between the studied groups

Paired-Samples t-test

* Statistically significant at ***P*** ≤ 0.05

### Comparison of Nitrate Consumption Per Week Between the Two Groups

There were no significant differences in the amount of nitrate consumption per week between the two groups before treatment (***P*** > 0.05), as shown in Table 8. In the TMZ group, the amount of nitrate consumption per week was significantly lower after than before treatment (***P*** < 0.05). The placebo group had a significant difference in the amount of nitrate consumption per week after than before treatment (***P*** < 0.05). After treatment, the TMZ group had a substantially lower amount of nitrate consumption per week compared to the placebo group (***P*** < 0.05).

**Table 8:** Comparison of nitrate consumption per week between the two groups

Nitrate consumption per week	TMZ group (*n* = 24)	Placebo group (*n* = 24)	**P**
Baseline			
Mean ± SD	3.9 ± 2.2	3.9 ± 2.1	0.788
After 3 months			
Mean ± SD	2.4± 1.2	3.3 ± 1.3	0.005^*^
**P**_**1**_	0.004^*^	0.036^*^	

**P**, ***P***-value for comparing between the studied groups

Paired-samples t-test

* Statistically significant at ***P*** ≤ 0.05

## Discussion

ICM, which remains a considerable burden in developing countries [5], has been the leading cause of heart failure (HF), accounting for approximately 60% of cases globally [18, 19]. HF has been associated with substantially higher rates of mortality and morbidity in humans [18, 20].

Evidence has shown that enhancement in HF functional class [5, 21], systolic function and functional capacity without changes in cardiac perfusion [22], wall motion score index at rest [5, 23], LV end-diastolic volume [5, 24], and the inflammatory process measured using CRP levels in the blood was the major benefit of TMZ [5, 21, 25]. A long-term study (24 months of follow-up) investigating the impact of TMZ on 200 ICM patients similar to those included herein revealed a significant decrease in the frequency of angina episodes per week (***P*** < 0.01) and correspondingly a decrease in sublingual nitroglycerin (glyceryl trinitrate) tablet intake per week [5, 26].

Novel knowledge regarding myocardial ischemia, a multifactorial disease, has been discovered in recent years [27, 28]. In addition to the vascular mechanisms of atherosclerotic CAD, non-vascular causes, such as cardiac energy metabolism disorders and variations in blood rheology due to platelet activation and/or inflammation, would likely be included among the mechanisms responsible for myocardial ischemic syndromes in the near future [27, 29, 30].

HF can further influence metabolic changes. Therefore, targeting cardiac metabolism in patients with ischemic heart disease can prevent the poor prognosis of LV function and prevent progression to HF. For these purposes, several trials have indicated the benefits of TMZ, a free fatty acid oxidation inhibitor that converts cardiac and muscle metabolism to glucose utilization. A retrospective cohort study examining the data from 669 participants with congestive HF (CHF) showed that 362 patients were on TMZ due to recurrent symptoms after taking all traditional CHF medications, whereas the remaining patients were treated with traditional CHF medications. As an additional therapy to other conventional CHF drugs, TMZ has improved mortality and event-free survival in CHF patients [31]. Given its mechanism of action, TMZ has been shown to have cardioprotective effects in angina patients and LV dysfunction, as well as those undergoing revascularization procedures, without any relevant side effects. TMZ, including other antianginal medications, have successfully improved exercise tolerance, delayed angina symptoms initiation, and extended the time to 1-mm ST-segment depression through exercise relative to placebo in a randomized placebo-controlled study involving 166 participants who were reluctant to use nitrates or beta-blockers. The aforementioned study also confirmed that TMZ could be used in conjunction with hemodynamic agents given that the rate pressure product was unchanged [32, 33].

A prospective double-blind, controlled trial that sought to investigate the impact of the preoperative use of TMZ on the biochemical parameters of myocardial injury, including myoglobin, troponin T, creatine kinase (CK), and creatine kinase muscle and brain (CK-MB) during CABG, found that the TMZ group had significantly lower levels of biochemical markers after surgery compared to the placebo group, which suggested that TMZ offered more protection of the myocardium [33, 34].

The ESC recommendations for the diagnosis and treatment of chronic coronary syndromes suggested that TMZ can be used as first-line therapy to minimize angina incidence and increase exercise tolerance in subjects with low baseline heart rate and low blood pressure [15]. Considering its lack of impact on heart rate or arterial blood pressure, the neutral hemodynamic profile of TMZ makes it an appealing choice for patients as well as clinicians [32]. Conversely, several studies have shown that TMZ (applied chronically in vivo or acutely in vitro) had no impact on cardiac fatty acid and carbohydrate oxidation, implying that therapeutic effects of TMZ were more likely due to an intracardiac mechanism that has yet to be identified [35].

The therapeutic effects of TMZ on cardiac fibrosis in ICM patients must be verified conclusively. Our study indicated that the addition of MR TMZ to traditional ICM therapies improved the patients’ vascular endothelial function, reduced the inflammatory process, and reduced the risk of cardiac fibrosis—an added clinical effect. Our research also reported that TMZ improved certain echocardiographic parameters.

ET-1 is a vascular contracting substance synthesized by vascular endothelial cells, which is important for the normal function of vascular smooth muscle cells [36]. The increased ET-1 production during cardiac ischemia and reperfusion further exacerbates the problem [37].

In our study, the combined regimen in the TMZ group effectively and significantly improved ET-1 levels (***P*** < 0.05). This may be attributed to the shifting of energy substrates away from fatty acid metabolism and toward glucose metabolism caused by TMZ [17].

The findings of the current study are consistent with those presented in a randomized, double-blind, crossover study where those who received TMZ exhibited lower endothelin-1 release 15 days after treatment relative to those who received placebo [4, 38]. Other studies have demonstrated a reduction in ET-1 serum levels in patients with diabetes undergoing TMZ treatment, both after short-term (2 weeks) and long-term (6 months) therapy; however, no decrease was detected in the placebo group [4, 17].

Inflammation is a key factor in the development of ventricular remodeling. Inflammatory cytokines, such as IL-1, IL-6, and TNF-α, have been shown to increase dramatically after myocardial infarction and are involved in future LV remodeling [39].

The current study found that while both regimens can effectively improve TNF-α levels, the level of improvement was significantly better in the TMZ group than in the placebo group, suggesting that TMZ can effectively reduce inflammatory stress in patients with ICM.

Consistent with our results, the TMZ group exhibited stable CRP plasma concentrations, whereas the placebo group experienced a significant increase in CRP levels throughout the 18-month treatment duration [4, 21].

The interstitial proliferation of fibroblasts induces cardiac fibrosis, whereas prolonged extracellular matrix precipitation causes HF, arrhythmia, sudden cardiac death, and other severe complications. Pressure overload leads to ventricular remodelings, such as myocyte hypertrophy and interstitial fibrosis, when prolonged. Cardiac fibrosis remains a key factor in the progression from compensated ventricular hypertrophy to HF. Notably, CTGF plays a crucial role in the cardiac fibrosis process and has emerged as a novel therapeutic target in the treatment of fibrotic diseases [12]. CTGF is a member of the CCN family of multifunctional matricellular proteins that play a role in initiating fibrosis in a variety of organs and tissues, including the heart. CTGF, which is also a key mediator of the profibrotic cytokine transforming growth factor- (TGF-) signaling pathway, stimulates the proliferation of fibroblasts and increases the contents of the extracellular matrix. CTGF is a “bystander” marker that has the potential to influence the fibrosis process indirectly [11]. Limited data have been available regarding the effects of TMZ on myocardial fibrosis that occurs after ischemia and causes LV dysfunction [14]. Assessment of CTGF as an initiator biomarker of cardiac fibrosis is suitable for NYHA II patients who are in the early stages of disease progression.

Our study revealed that while both groups showed a significant decrease in the CTGF levels, the TMZ group showed better improvement. Our results confirmed that as adjuvant therapy, TMZ can effectively improve the cardiac fibrosis process in patients with ICM. The mechanism by which TMZ exerts its effects may be linked to decreased collagen synthesis, CTGF expression in cardiac fibroblasts, nicotinamide adenine dinucleotide phosphate-oxidase levels, and production of reactive oxygen species [8, 12].

After analyzing the myocardial function of the patients, our results showed that LVEF was increased significantly, whereas both LVESD and LVEDD were significantly decreased in the TMZ group. Notably, new findings observed herein suggested that LV dysfunction in ICM patients is mediated by changes in substrate metabolism. TMZ improves heart metabolism by altering the preferred energy substrate from fatty acid to glucose oxidation, thereby potentially playing an important role in improving myocardial function [23]. The drug’s effects on maintaining the integrity of cell membranes [2], as well as mitochondrial structure and function, may explain the improvement in the LVEF [2, 40]. The increase in glucose oxidation caused by TMZ can improve contractility and microvascular function by promoting glycolytic ATP resynthesis. TMZ treatment may also increase the energy metabolism of chronically hibernated cells, making them more efficient at generating contractile activity, limiting further LV function decline [5]. Although this difference may not be clinically significant, a better effect can be expected with a longer treatment period. However, clinical improvement was detected in the form of a reduction in the number of angina attacks per week and nitrate consumption in the TMZ group when compared with the placebo group.

Our findings showed that ET-1, CTGF, and TNF-α were a bit significantly correlated with echocardiographic indices in the TMZ group (***P*** > 0.05). This may be explained by the small non-clinically significant changes in LVEF, LVESD, and LVEDD. However, with longer treatment duration, a better correlation may be expected.

### Drug Tolerance

TMZ had been well tolerated throughout the study, with none of the participants needing to cease administration. The established pharmacological actions of TMZ are supported by its neutral hemodynamic impact without significantly modifying systolic or diastolic BP or heart rate [41, 42].

### Study Limitations

One major limitation of the current study is the limited sample size, with only 48 patients having been included (24 in each group). At the 3-month follow-up examination, the effects of TMZ were detected. To further demonstrate the cardioprotective function of TMZ, more studies with larger sample size and longer follow-ups are required. Furthermore, cardiac fibrosis is best measured with cardiac magnetic resonance (MR) imaging, considering that CTGF is an indirect method. In addition, more than two indicators were needed to jointly corroborate cardiac fibrosis, vascular endothelial function, and the inflammatory process. While there are improvements in markers of fibrosis and potential echocardiographic indices of ventricular function, functional assessments are required. As such, we consider this study an exploratory trial.

## Conclusion

Adding TMZ to conventional medications for ICM treatment can be of clinical value given its ability to improve vascular endothelial function, hinder inflammatory process progression, and protect myocardial cells by improving some echocardiographic indices, such as LVEF, LVESD, and LVEDD. This study demonstrated that TMZ can effectively inhibit the cardiac fibrosis process in clinical settings by assessing CTGF as an initiator biomarker of cardiac fibrosis.

## Data Availability

The Authors can confirm that all relevant data are included in the article and/or its supplementary information files.

## References

[1] Malakar AK, Choudhury D, Halder B, Paul P, Uddin A, Chakraborty S (2019). A review on coronary artery disease, its risk factors, and therapeutics. J Cell Physiol..

[2] Bhandari B, Rodriguez BSQ, Masood W. Ischemic cardiomyopathy. In: StatPearls [Internet]. Treasure Island: StatPearls; 2020.

[3] Sekulic M, Zacharias M, Medalion B (2019). Ischemic cardiomyopathy and heart failure: consideration for fibromuscular dysplasia with intimal fibroplasia of coronary arteries. Circ Heart Fail..

[4] Bertomeu-Gonzalez V, Bouzas-Mosquera A, Kaski JC (2006). Role of trimetazidine in management of ischemic cardiomyopathy. Am J Cardiol..

[5] Momen A, Ali M, Karmakar PK (2016). Effects of sustained-release trimetazidine on chronically dysfunctional myocardium of ischemic dilated cardiomyopathy - six months follow-up result. Indian Heart J..

[6] Chrusciel P, Rysz J, Banach M (2014). Defining the role of trimetazidine in the treatment of cardiovascular disorders: some insights on its role in heart failure and peripheral artery disease. Drugs..

[7] Mann DL. Heart failure: a companion to Braunwald’s heart disease E-book 2nd ed: Elsevier Health Sciences; 2010.

[8] McCarthy CP, Mullins KV, Kerins DM (2016). The role of trimetazidine in cardiovascular disease: beyond an anti-anginal agent. Eur Heart J Cardiovasc Pharmacother..

[9] Fragasso G, Anastasia G, Monaca G, Pinto G. Clinical benefits of targeting cardiac cells directly with trimetazidine in patients with coronary disease and diabetes. Heart Metab. 2017;73:24–8.

[10] Dézsi CA (2016). Trimetazidine in practice: review of the clinical and experimental evidence. Am J Ther..

[11] Ding Y, Wang Y, Zhang W (2020). Roles of biomarkers in myocardial fibrosis. Aging Dis..

[12] Liu X, Gai Y, Liu F (2010). Trimetazidine inhibits pressure overload-induced cardiac fibrosis through NADPH oxidase–ROS–CTGF pathway. Cardiovasc Res..

[13] Zhao Y, Li S, Quan E (2019). Trimetazidine inhibits cardiac fibrosis by reducing reactive oxygen species and downregulating connective tissue growth factor in streptozotocin-induced diabetic rats. Exp Ther Med..

[14] Chen Z, Zhang N, Zhang Z, Zhang G, Zhang B (2020). Connective tissue growth factor: from molecular understandings to drug discovery. Front Cell Dev Biol..

[15] Knuuti J, Wijns W, Saraste A (2020). 2019 ESC Guidelines for the diagnosis and management of chronic coronary syndromes. Eur Heart J.

[16] Kosaraju A, Goyal A, Grigorova Y, Makaryus AN. Left ventricular ejection fraction. In: StatPearls. StatPearls Publishing; 2017. 29083812

[17] Fragasso G, Piatti P, Monti L (2003). Short- and long-term beneficial effects of trimetazidine in patients with diabetes and ischemic cardiomyopathy. Am Heart J..

[18] Wen J, Ma X, Zhang L (2018). Therapeutic efficacy and safety of Shexiang Baoxin Pill combined with trimetazidine in elderly patients with heart failure secondary to ischaemic cardiomyopathy: a systematic review and meta-analysis. Medicine..

[19] Isomura T, Hirota M, Hoshino J (2013). Strategy of treatment for ischemic cardiomyopathy. J Jpn Coron Assoc..

[20] Mentz RJ, Broderick S, Shaw LK, Chiswell K, Fiuzat M, O’Connor CM. Persistent angina pectoris in ischaemic cardiomyopathy: increased rehospitalization and major adverse cardiac events. Eur J Heart Fail. 2014;16:854–60. 10.1002/ejhf.130. 10.1002/ejhf.130 PMC5702887 24975128

[21] Zhao P, Zhang J, Yin X-G (2013). The effect of trimetazidine on cardiac function in diabetic patients with idiopathic dilated cardiomyopathy. Life Sci..

[22] Belardinelli R, Cianci G, Gigli M, Mazzanti M, Lacalaprice F (2008). Effects of trimetazidine on myocardial perfusion and left ventricular systolic function in type 2 diabetic patients with ischemic cardiomyopathy. J Cardiovasc Pharmacol..

[23] Hu B, Li W, Xu T, Chen T, Guo J (2011). Evaluation of trimetazidine in angina pectoris by echocardiography and radionuclide angiography: A meta-analysis of randomized, controlled trials. Clin Cardiol..

[24] Zhang L, Lu Y, Jiang H (2012). Additional use of trimetazidine in patients with chronic heart failure: a meta-analysis. J Am Coll Cardiol..

[25] Di Napoli P, Di Giovanni P, Gaeta MA, Taccardi AA, Barsotti A (2007). Trimetazidine and reduction in mortality and hospitalization in patients with ischemic dilated cardiomyopathy: a post hoc analysis of the Villa Pini d’Abruzzo Trimetazidine Trial. J Cardiovasc Pharmacol..

[26] El-Kady T, El-Sabban K, Gabaly M, Sabry A, Abdel-Hady S (2005). Effects of trimetazidine on myocardial perfusion and the contractile response of chronically dysfunctional myocardium in ischemic cardiomyopathy: a 24-month study. Am J Cardiovasc Drugs..

[27] Marzilli M, Crea F, Morrone D (2020). Myocardial ischemia: from disease to syndrome. Int J Cardiol..

[28] Crea F, Camici PG, Bairey Merz CN (2014). Coronary microvascular dysfunction: an update. Eur Heart J..

[29] Fillmore N, Mori J, Lopaschuk GD (2014). Mitochondrial fatty acid oxidation alterations in heart failure, ischaemic heart disease and diabetic cardiomyopathy. Br J Pharmacol..

[30] Martins-Marques T, Catarino S, Marques C (2015). Heart ischemia results in connexin43 ubiquitination localized at the intercalated discs. Biochimie..

[31] Fragasso G, Rosano G, Baek SH (2013). Effect of partial fatty acid oxidation inhibition with trimetazidine on mortality and morbidity in heart failure: results from an international multicentre retrospective cohort study. Int J Cardiol..

[32] Chazov EI, Lepakchin VK, Zharova EA (2005). Trimetazidine in Angina Combination Therapy--the TACT study: trimetazidine versus conventional treatment in patients with stable angina pectoris in a randomized, placebo-controlled, multicenter study. Am J Ther..

[33] Marzilli M, Vinereanu D, Lopaschuk G (2019). Trimetazidine in cardiovascular medicine. Int J Cardiol..

[34] Iskesen I, Kurdal AT, Eserdag M, Cerrahoglu M, Sirin BH, editors. Trimetazidine may protect the myocardium during cardiac surgery. Heart Surg Forum. 2009;12:E175–9. 10.1532/HSF98.20081133 19546072

[35] Cavar M, Ljubkovic M, Bulat C (2016). Trimetazidine does not alter metabolic substrate oxidation in cardiac mitochondria of target patient population. Br J Pharmacol..

[36] Sandri M, Viehmann M, Adams V (2016). Chronic heart failure and aging–effects of exercise training on endothelial function and mechanisms of endothelial regeneration: results from the Leipzig exercise intervention in chronic heart failure and aging (LEICA) study. Eur J Prev Cardiol..

[37] Skovsted GF, Kruse LS, Berchtold LA, Grell A-S, Warfvinge K, Edvinsson L (2017). Myocardial ischemia-reperfusion enhances transcriptional expression of endothelin-1 and vasoconstrictor ETB receptors via the protein kinase MEK-ERK1/2 signaling pathway in rat. PLoS One..

[38] Monti LD, Setola E, Fragasso G (2006). Metabolic and endothelial effects of trimetazidine on forearm skeletal muscle in patients with type 2 diabetes and ischemic cardiomyopathy. Am J Physiol Endocrinol..

[39] Zhou X, Li C, Xu W, Chen J (2012). Trimetazidine protects against smoking-induced left ventricular remodeling via attenuating oxidative stress, apoptosis, and inflammation. PloS One..

[40] Wu L, Luan Y, Li Y (2020). Effects of trimetazidine on ventricular remodeling in coronary artery disease patients with left ventricular hypertrophy: the rationale and design of a randomized controlled trial. BMC Cardiovasc Disord..

[41] Kanorskii SG, Smolenskaya NV (2016). Triple antianginal combinations in the treatment of elderly and senile patients with stable angina. Ter Arkh.

[42] Jatain S, Kapoor A, Sinha A (2016). Metabolic manipulation in dilated cardiomyopathy: assessing the role of trimetazidine. Indian Heart J..

